# *Arabidopsis *ETO1 specifically interacts with and negatively regulates type 2 1-aminocyclopropane-1-carboxylate synthases

**DOI:** 10.1186/1471-2229-5-14

**Published:** 2005-08-10

**Authors:** Hitoshi Yoshida, Masayasu Nagata, Koji Saito, Kevin LC Wang, Joseph R Ecker

**Affiliations:** 1 Department of Rice Research, National Agricultural Research Center, Jo-etsu, Niigata 943–0193, Japan; 2 Department of Low-Temperature Sciences, National Agricultural Research Center for Hokkaido Region, Sapporo, Hokkaido 062–8555, Japan; 3 Department of Physiology and Quality Science, National Institute of Vegetable and Tea Science, Ano, Mie 514–2392, Japan; 4 Plant Biology Laboratory, The Salk Institute for Biological Studies, La Jolla, California 92037, U.S.A; 5 Present address: Institute of Plant and Microbial Biology, Academia Sinica, Nankang, Taipei 115, Taiwan

## Abstract

**Background:**

In *Arabidopsis*, ETO1 (ETHYLENE-OVERPRODUCER1) is a negative regulator of ethylene evolution by interacting with AtACS5, an isoform of the rate-limiting enzyme, 1-aminocyclopropane-1-carboxylate synthases (ACC synthase or ACS), in ethylene biosynthetic pathway. ETO1 directly inhibits the enzymatic activity of AtACS5. In addition, a specific interaction between ETO1 and AtCUL3, a constituent of a new type of E3 ubiquitin ligase complex, suggests the molecular mechanism in promoting AtACS5 degradation by the proteasome-dependent pathway. Because orthologous sequences to ETO1 are found in many plant species including tomato, we transformed tomato with *Arabidopsis ETO1 *to evaluate its ability to suppress ethylene production in tomato fruits.

**Results:**

Transgenic tomato lines that overexpress *Arabidopsis ETO1 *(*ETO1*-OE) did not show a significant delay of fruit ripening. So, we performed yeast two-hybrid assays to investigate potential heterologous interaction between ETO1 and three isozymes of ACC synthases from tomato. In the yeast two-hybrid system, ETO1 interacts with LE-ACS3 as well as AtACS5 but not with LE-ACS2 or LE-ACS4, two major isozymes whose gene expression is induced markedly in ripening fruits. According to the classification of ACC synthases, which is based on the C-terminal amino acid sequences, both LE-ACS3 and AtACS5 are categorized as type 2 isozymes and possess a consensus C-terminal sequence. In contrast, LE-ACS2 and LE-ACS4 are type 1 and type 3 isozymes, respectively, both of which do not possess this specific C-terminal sequence. Yeast two-hybrid analysis using chimeric constructs between LE-ACS2 and LE-ACS3 revealed that the type-2-ACS-specific C-terminal tail is required for interaction with ETO1. When treated with auxin to induce *LE-ACS3*, seedlings of *ETO1*-OE produced less ethylene than the wild type, despite comparable expression of the *LE-ACS3 *gene in the wild type.

**Conclusion:**

These results suggest that ETO1 family proteins specifically interact with and negatively regulate type 2 ACC synthases. Our data also show that *Arabidopsis *ETO1 can regulate type 2 ACS in a heterologous plant, tomato.

## Background

Ethylene is a simple gas that acts as a plant hormone; it controls various processes in the plant life cycle, including seed germination, root hair development, root nodulation, flower senescence, abscission, and fruit ripening [1]. Ethylene also is synthesized in response to stresses such as pathogen attack, wounding, hypoxia, ozone, chilling, and freezing [2]. These responses are controlled through integration of the pathways for ethylene biosynthesis, perception, and signal transduction. The ethylene biosynthesis and signalling pathways have been well characterized with regard to physiology, biochemistry, molecular biology, and genetics. Studies of *Arabidopsis thaliana *revealed a universally conserved set of components in the ethylene signaling pathway for all plants. Ethylene is perceived by ethylene receptor family ETR1, ETR2, EIN4, ERS1, ERS2 [3-6] and their assembly requires the RAN1 copper transporter [7]. The signal is further transduced by the signaling pathway components CTR1, a Raf-like MAPKKK [8,9], EIN2, an Nramp-related integral membrane protein [10], and a cascade of transcriptional regulators EIN3/EILs [11], ERF1 [12], EDF1-4 [13]. The proteolysis of EIN3/EIL proteins by ethylene-regulated EBF1/2 F-box proteins plays a key role in regulation of all known responses to the hormone [14,15]. A MAPK (including AtMPK6) cascade was proposed to be involved in ethylene signaling [16], however, a recent study showed that AtMPK6 is not involved in ethylene signaling but instead involved in the regulation of ethylene biosynthesis through AtACS6 [17,18]. Other yet unidentified components are thought to be involved as well [19,20].

The biosynthetic pathway of ethylene has been studied in detail, and genes encoding the two key enzymes have been cloned and characterized [21-25]. 1-aminocyclopropane-1-carboxylate (ACC) synthase (ACS) converts *S*-adenosyl-L-methionine (SAM) to ACC and then, ACC oxidase (ACO) produces ethylene through oxidization of ACC. Generally, the reaction catalyzed by ACS is a rate-limiting step [26].

Both ACS and ACO are encoded by gene families in many plant species. In the case of tomato (*Lycopersicon esculentum *Mill.), there are at least 10 *ACS *[27] and four *ACO *genes [28]. The members of *ACS *and *ACO *gene families are differentially expressed in development or in response to stimuli such as germination, leaf senescence and flower abscission, fruit ripening, wounding, flooding, exposure to ozone, touch, hormone treatment, and pathogen attack [28-33]. Recent findings suggest that posttranscriptional regulation is an important aspect of the control of *ACS *expression [22,34-38]. Pharmacological and molecular biological studies have suggested that phosphorylation is involved in the regulation of ACS activity [34,39]. A tomato ACS, LE-ACS2, is phosphorylated in the C-terminal region and this modification appears to be involved in the posttranslational regulation of the enzyme [40]. However, the molecular mechanisms by which plants regulate ethylene biosynthesis at the posttranscriptional or posttranslational levels remain unclear.

Recent studies of the *Arabidopsis ethylene-overproducer *mutants *(eto1*,*eto2-1*,*eto3) *[36-38,41] revealed mechanisms underlying the posttranslational regulation of ethylene biosynthesis. The *eto *mutants constitutively display the ethylene-evoked triple response phenotypes in the absence of exogenously applied hormone; they can be distinguished from *ctr1 *mutants, which also displays the triple response in the absence of ethylene, because the phenotypes of *eto *mutants are suppressed by inhibitors of ethylene biosynthesis and action [8,42]. Therefore, the *eto *mutants are likely impaired in either the regulators or the structural enzymes of ethylene biosynthesis. *eto1 *is a recessive mutation that results in an approximately 10-fold ethylene overproduction in etiolated seedlings compared to wild-type plants [42], whereas *eto2-1 *and *eto3 *are dominant mutations that cause 20- and 100-fold increases of ethylene biosynthesis, respectively, in etiolated seedlings [8]. The *eto2-1 *and *eto3 *were identified as mutations within the closely related *AtACS5 *and *AtACS9 *genes, respectively. These mutations cause alterations of the C-terminal amino acid sequences of each protein [36,38]. Furthermore, both *eto1 *and *eto2-1 *mutations increase the stability of the AtACS5 protein [38], suggesting that the C-termini of some ACC synthases are involved in posttranslational regulation/processing or stability of the ACS proteins mediated by ETO1. Also another study showed that AtMPK6 phosphorylates the C-terminus of AtACS6 to stabilize the isozyme by a yet unknown mechanism [17].

The molecular mechanism(s) regulating the activity and stability of AtACS5 was revealed by identification of the ETO1 protein [41]. ETO1 is a member of a novel plant-specific protein family with three distinct protein-protein interaction motifs, namely the BTB domain in its N-terminus and the TPR motifs together with a coiled-coil motif in its C-terminus. The C-terminal TPR domain interacts with AtACS5 and the N-terminal BTB domain interacts with AtCUL3, a constituent of E3 ubiquitin ligase complexes in which ETO1 is proposed to serve as a substrate-specific adaptor protein. ETO1 inhibits the enzyme activity of AtACS5 and targets this protein for degradation in a proteasome-dependent manner. Other studies also suggest that proteolysis is involved in regulation of ethylene biosynthesis [43,44].

ETO1 has two paralogs in *Arabidopsis*, EOL1 and EOL2 (ETO1-LIKE), both of which also interact with and inhibit the activity of AtACS5. Although ETO1-related sequences are found in many other plant species including tomato, no studies have examined whether members of the ETO1 protein family can interact with and regulate ACC synthases other than AtACS5. In this study, using a heterologous system in tomato, we demonstrate that ETO1 specifically interacts with a subfamily of ACC synthases, namely type 2 ACC synthases including AtACS5, AtACS9 and LE-ACS3, but not with other types of ACC synthases including AtACS6, LE-ACS2 and LE-ACS4. We also show that constitutive expression of *ETO1 *results in posttranscriptional suppression of a type 2 ACC synthase, LE-ACS3, in transgenic tomato. These results suggest that members of the ETO1 protein family are components in the negative regulation of type 2 ACC synthases in the plant kingdom.

## Results

### Fruit ripening is not altered in transgenic tomato plants that overexpress ETO1

**Figure 1:**
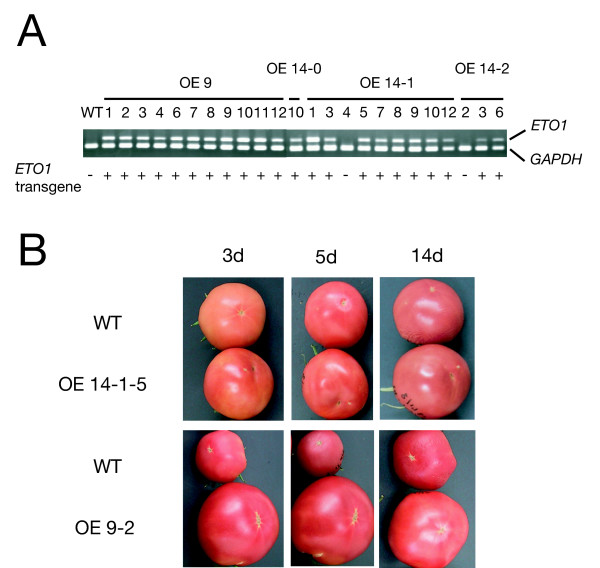
**Transgenic tomato plants that overexpress the *ETO1 *transgene did not show altered fruit ripening**. A. Expression of the *ETO1 *transgene in leaves of T_1 _individuals. Expression of *ETO1 *was analyzed by RT-PCR. *GAPDH *was used as an internal control. One microgram of total RNA was used for each reaction. WT: wild type (*Lycopersicon esculentum *cv. Shu-gyoku); OE 9, OE 14-0, OE 14-1, OE 14-2 represent independent T_0 _transformants. Numbers under the horizontal lines represent T_1 _segregating individuals derived from the corresponding T_0 _parents. Genotyping for the *ETO1 *transgene was also performed by PCR and shown under the photograph. B. Representative phenotype of two independent T_1 _progenies of *ETO1 *transgenic tomato (line #14-1-5 and #9-2) and wild type (WT). Fruits were harvested at breaker stage and allowed to ripen for further days as indicated.

Orthologous sequences to ETO1 are found in many plant species including tomato [HY and JRE, unpublished data]. These orthologs share high similarity with ETO1 in their TPR domains that are thought to be involved in specific interaction with ACC synthases [41]. Actually, two ETO1 paralogs of *Arabidopsis*, EOL1 and EOL2, interact with AtACS5 in yeast cells and suppress its activity in *Escherichia coli*. In tomato, ethylene is a critical regulator of fruit ripening [45], and LE-ACS2 and LE-ACS4 have been shown to be involved in this process [46]. So, there is a possibility that any of the ETO1 ortholog(s) in tomato interact with and regulate these ACS isozymes. Because we expected heterologous interaction between *Arabidopsis *ETO1 and LE-ACS2 or LE-ACS4, leading to suppression of ethylene biosynthesis in tomato fruits and retardation of its development, we introduced the *Arabidopsis ETO1 *cDNA controlled by the CaMV 35S promoter into tomato (*L. esculentum *cv. Shu-gyoku) via *Agrobacterium*-mediated transformation. Transformants were selected on kanamycin-containing medium and verified using the polymerase chain reaction (PCR) (see methods). Expression of the *ETO1 *transgene in the T_1 _segregating individuals was detected by reverse transcription (RT)-PCR assays. All of the T_1 _lines harbouring the transgene showed expression of the exogenous *ETO1 *gene (Fig. 1A). Twenty-two T_1 _individual *ETO1*-overexpressing (*ETO1*-OE) lines were derived from four independent T_0 _lines (Fig. 1A). However, all of the fruits of *ETO1*-OE lines developed a full red color over the same time course as wild-type plants (Fig. 1B). These results suggest that ETO1 may not suppress the two major ACS isozymes in ripening tomato fruit, LE-ACS2 and LE-ACS4, because there is no noteworthy interaction of ETO1 with either LE-ACS2 or LE-ACS4.

### ETO1 specifically interacts with LE-ACS3, but not with LE-ACS2 or LE-ACS4, in yeast cells

**Figure 2:**
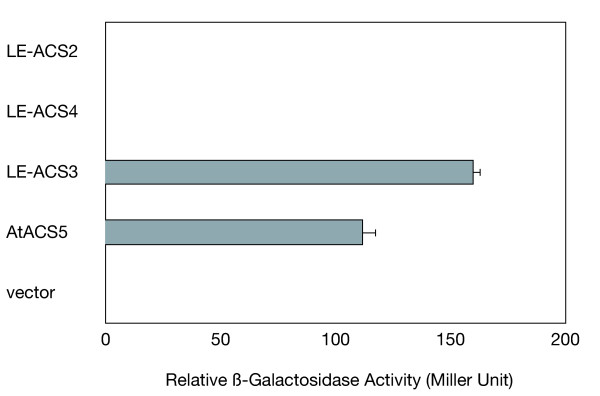
***Arabidopsis *ETO1 interacts with LE-ACS3 but not with LE-ACS2 or LE-ACS4 in yeast**. Interaction between *Arabidopsis *ETO1 (in pAS2) and ACC synthases (in pACT2) from tomato (LE-ACS2, LE-ACS3, and LE-ACS4) were analyzed in the yeast two-hybrid system. AtACS5 from *Arabidopsis *was used as a positive control representing a strong interaction partner with ETO1. pACT2 vector was used as a negative control. Three independent original transformants were analyzed for each combination. Means ± SE (*n *= 3) are indicated.

To investigate the potential interaction between the *Arabidopsis *ETO1 and ACC synthases of tomato, we performed yeast two-hybrid assays between ETO1 and three ACS isozymes of tomato, LE-ACS2, LE-ACS3, and LE-ACS4. As described above, LE-ACS2 and LE-ACS4 are major ACS isozymes involved in ripening tomato fruits. LE-ACS3 is induced by auxin and flooding [46,47]. In the yeast two-hybrid assay, whereas LE-ACS3 showed a strong interaction with ETO1, at a level comparable to that of AtACS5, neither LE-ACS2 nor LE-ACS4 interacted with ETO1 (Fig. 2). These results suggest that ETO1 may have a preference for a type of ACS isozymes that include LE-ACS3 and AtACS5.

**Figure 3:**
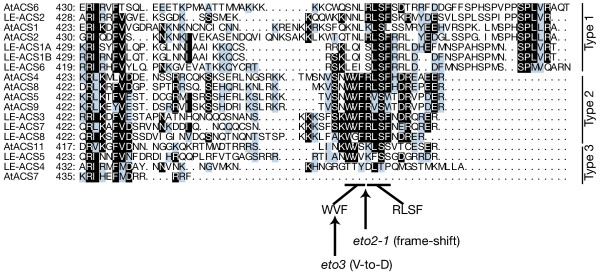
**Classification based on C-termini of ACC synthases of tomato and *Arabidopsis***. ACC synthases can be grouped into three distinct types based on the similarity of their C-terminal amino acid sequences. The C-terminal amino acid sequences of ACC synthases were aligned using the CLASTALW program, drawn using MacBoxShade, and grouped into three distinct types. Eight ACC synthases from *Arabidopsis *(AtACS1, AtACS2, AtACS4, AtACS5, AtACS6, AtACS7, AtACS8, AtACS9, and AtACS11; AtACS3 is thought to be a pseudogene, and AtACS10 and AtACS12 are aminotransferases) and nine from tomato (LE-ACS1A, LE-ACS1B, LE-ACS2, LE-ACS3, LE-ACS4, LE-ACS5, LE-ACS6, LE-ACS7, and LE-ACS8) were aligned. Identical amino acids and conservative changes are indicated by reversed and shaded characters, respectively. The WVF and RLSF motifs and mutation sites for *eto2-1 *and *eto3 *are indicated.

To investigate this possibility, we focused on the C-termini of various ACC synthases from *Arabidopsis *and tomato as potential targets of ETO1. C-terminal amino acid sequences of ACC synthases are the least-conserved portion of these proteins [26,48]. However, there is one small conserved motif, RLSF (arginine [R] – leucine [L] – serine [S] – phenylalanine [F]), in the C-termini of several ACC synthases [27,38,40]. A classification of ACC synthases based on the similarity of DNA sequences was reported by Oetiker et al. [35], and phylogenetic classifications based on the similarity of amino acid sequences of *Arabidopsis *ACC synthases were also devised [17,49]. Apart from these classifications, based on the C-terminal consensus motif mentioned above, we further classified the ACS isozymes of tomato and *Arabidopsis *into three types as shown in Fig. 3: (1) isozymes with long tails (23–27 amino acids) after the RLSF consensus sequence (like LE-ACS2); (2) isozymes that possess the 'WVF (tryptophan [W] – valine [V] – phenylalanine [F])' consensus sequence just before the RLSF, have short tails (5–8 amino acids) rich in arginine [R] and acidic amino acids (aspartic acid [D] or glutamic acid [E]) after the RLSF, and end with the 'ER (glutamic acid [E] – arginine [R])' consensus (like AtACS5 and LE-ACS3); and (3) isozymes lacking the RLSF consensus sequence (like AtACS7 and LE-ACS4). AtACS3, AtACS10, and AtACS12 are not included in this alignment because *AtACS3 *is a pseudogene, and AtACS10 and 12 are not ACC synthases but aminotransferases [27]. Hereafter, we use the word "type" instead of "class" to avoid confusion between former sequence-based classifications and our functional classification based on C-terminal motifs and the capacity to bind ETO1/EOL proteins.

Although the lengths of the C-terminal tails of AtACS11, LE-ACS4, and LE-ACS5 are comparable to those of type 2 isozymes, they lack the RLSF motif and the R/D/E-rich region. Therefore, we classified these three isozymes as type 3 isozymes along with AtACS7, which has a much shorter C-terminal tail. Both AtACS5 and LE-ACS3, which interact strongly with ETO1, belong to type 2, whereas LE-ACS2 belongs to type 1 and LE-ACS4 to type 3. Because we previously showed that the C-terminus of AtACS5 is a target of ETO1, this raises the possibility that the C-termini of type 2 ACC synthases contain some specific features necessary for the interaction with ETO1 that may also be conserved in other plants.

### ETO1 does not interact with a series of C-terminal deletion mutants of LE-ACS2 in yeast

As described above, one notable feature of type 2 ACC synthases is the length of the C-terminal tail after the RLSF motif. Type 1 ACC synthases possess longer C-terminal tails after the RLSF than type 2, whereas type 3 isozymes lack the RLSF. Furthermore, the C-terminus of ACS appears to be proteolyzed *in vivo *[50]. Although another study showed that C-terminal truncation of a type 1 enzyme, LE-ACS2, does not occur *in vivo *[40], other type 1 ACC synthases may have their C-termini truncated to an "optimal length" (i.e., length comparable to type 2) and may then become capable of interacting with ETO1 family proteins. Therefore, we examined the effect of altering the length of ACS protein C-terminal tails on their interaction with ETO1.

**Figure 4:**
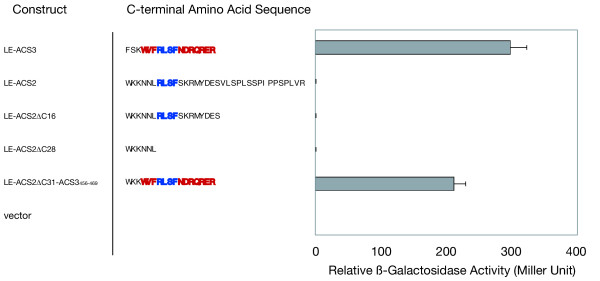
**Specific C-terminal amino acid sequence of type 2 ACC synthases is necessary for the interaction with ETO1 in the yeast two-hybrid system**. Interaction of ETO1 with C-terminal mutants of LE-ACS2 deleted or swapped with LE-ACS3 were analyzed by quantitative yeast two-hybrid assay. ETO1 was cloned into pAS2 and ACS mutants were cloned into pACT2 vectors, respectively. C-terminal amino acid sequences of each mutant are shown on the left. The WVF motif and the R/D/E-rich region derived from LE-ACS3 are shown in red. The RLSF motif common to both LE-ACS3 and LE-ACS2 is shown in blue. The pACT2 vector was used as a negative control. Three independent original transformants were analyzed for each combination. Means ± SE (*n *= 3) are indicated.

Two C-terminally truncated mutants of LE-ACS2 were constructed and the cDNA was cloned into pACT2 (see methods). LE-ACS2Δ16 contains a deletion of 16 amino acids from the C-terminal end; and as a result its C-terminus has a length comparable to native type 2 ACS proteins (Fig. 4). The other mutant, LE-ACS2Δ28, was trimmed to one amino acid upstream of the RLSF (Fig. 4). If a specific length of the C-terminal tail after the RLSF is necessary for the interaction with ETO1, then LE-ACS2Δ16 should interact with ETO1 whereas LE-ACS2Δ28 should not. However, neither of these truncated mutants was able to interact with ETO1 in the yeast two-hybrid assay (Fig. 4). These results suggest that the length of C-termini of type 1 ACS is not preventing ETO1 binding. Simply truncating the C-terminal tail of type 1 ACS to the length of type 2 isozymes is not sufficient to allow for interaction with ETO1 and implies that other factors are necessary for mediating this interaction.

### The C-terminal sequence specific to type 2 ACC synthases is required for the interaction with ETO1

Next we examined the effect of type-2-specific amino acid sequence around the RLSF on the interaction with ETO1. As mentioned above, type 2 ACC synthases have a specific C-terminal consensus sequence (i.e., the WVFRLSF motif followed by the R/D/E-rich region). The RLSF is conserved in both type 1 and type 2 ACC synthases, whereas the WVF motif and R/D/E-rich region are conserved only in type 2 ACC synthases in tomato and *Arabidopsis *(Fig. 3). In the *eto3 *mutant, the valine residue in this WVF motif is mutated to aspartic acid in AtACS9 [38], suggesting an important role of this small motif (Fig. 3). We replaced the C-terminal 31 amino acids of LE-ACS2 with amino acids 456–469 of LE-ACS3 (LE-ACS2ΔC31::LE-ACS3_456–469_). This chimeric ACS protein was sufficient to recover the strong interaction with ETO1 to a level comparable to LE-ACS3 (Fig. 4). These results strongly suggest that the C-terminal tail specific to type 2 ACS, comprising the WVFRLSF motif and the R/D/E-rich region, is necessary and sufficient for the interaction with ETO1.

### Overexpression of ETO1 suppresses auxin-induced ethylene biosynthesis in tomato seedlings

To investigate the interaction between ETO1 and LE-ACS3 *in planta*, we examined the induction of ethylene biosynthesis in etiolated tomato seedlings by a synthetic auxin, 2,4-D. Using T_2 _seedlings of a homozygous line of *ETO1*-OE (14-1-H2; not included in Fig. 1A), we measured ethylene evolution from auxin-treated wild-type and *ETO1 *transgenic tomato seedlings.

**Figure 5:**
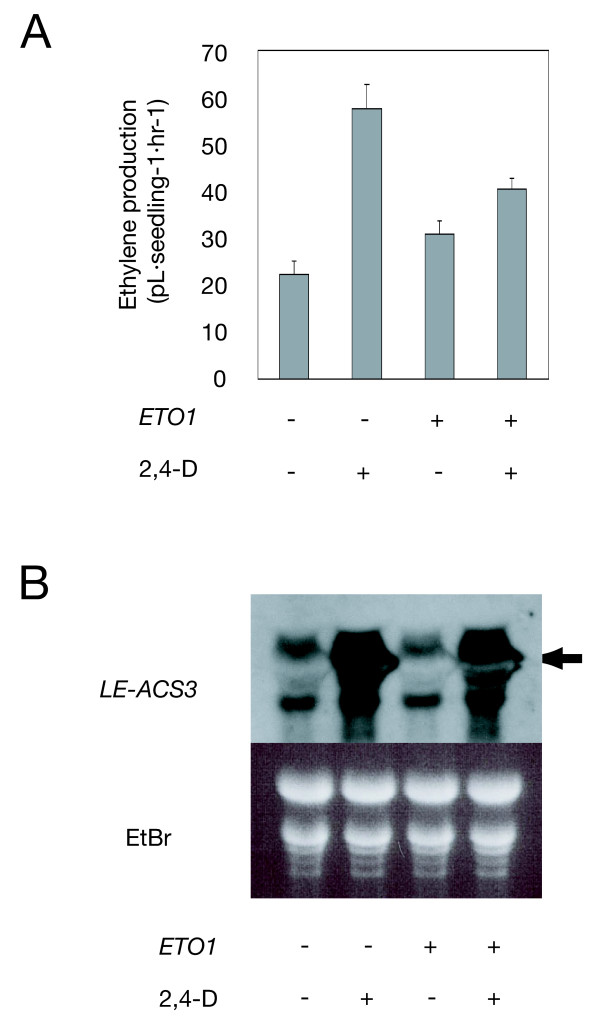
**ETO1 suppresses auxin-induced ethylene production in tomato**. A. Six-day-old etiolated seedlings of T_2 _homozygous lines, 14-1-H2 (*ETO1 *+/+) and 14-1-H4 (*ETO1 *-/-) were treated with 2,4-D (in 1% ethanol) at the final concentration of 100 μM for 1 d. Ethylene concentration in the headspace was measured by a gas chromatograph. Means ± SE (*n *= 8) are indicated. B, Induction of *LE-ACS3 *by 2,4-D. Total RNA (10 μg) from etiolated seedlings (*ETO1 *+/+ or -/-) with or without 2,4-D treatment was probed with *LE-ACS3*. Four individuals were used for each treatment. Arrowhead indicates the functional unspliced transcript size [51].

When 6-day-old etiolated wild-type seedlings were treated with 100 μM 2,4-D for 24 h, they produced 2.1-fold more ethylene than untreated seedlings (Fig. 5A). *LE-ACS3 *mRNA was induced by the 2,4-D treatment in the wild type (Fig. 5B). However, when the *ETO1*-OE transgenic plants were treated in this manner, they produced only 1.6-fold more ethylene when compared to untreated plants, despite *LE-ACS3 *mRNA being strongly induced to a level comparable to wild type (Fig. 5A and 5B). These results, together with those of the yeast two-hybrid assays, indicate that overexpressed ETO1 protein may suppress the ACS activity of LE-ACS3 by a direction, which results in a reduction in ethylene biosynthesis, same as the case of AtACS5 and cytokinin treatment in *Arabidopsis *[41].

## Discussion

### ETO1 interacts with and inhibits type 2 ACC synthases

In a previous study, we demonstrated that ETO1, a novel plant-specific BTB/TPR protein, negatively regulates an ethylene biosynthetic enzyme of *Arabidopsis *seedlings, AtACS5, via direct interaction [41]. However, whether the interaction and the regulatory effect are limited to AtACS5 and its paralogs/orthologs – or are common to the entire plant ACC synthase family – has not been clarified. In this study, we showed the interaction between ETO1 and ACS is restricted to type 2 ACS isozymes. We classified ACC synthases into three types based on their C-terminal amino acid sequences. All members of 'class I' ACC synthases of tomato (LE-ACS1A, LE-ACS1B, LE-ACS6) in the classification of Oetiker et al. [35] correspond to our type 1 ACC synthases, while both of their 'class II' (LE-ACS2 and LE-ACS4) and 'class III' (LE-ACS3 and LE-ACS5) contains members of our type 1 and type 3. This discrepancy may result from the different methods of the phylogenetic anlysis (i.e. partial DNA sequences [35] and C-terminal amino acid sequences [this paper]). On the other hand, our type 2 isozymes of *Arabidopsis *are grouped into two closely related phylogenetic branches in the group B ACC synthases [49], and the type 3 isozymes belong to the branches in the group B other than the 'type 2' branches. Also our type 1 isozymes correspond to the group A isozymes [49]. These coincidences may reflect the functional relevance of each type of the isozymes, and, indeed, functional heterodimerizations between closely related ACS isozymes have been demonstrated [49].

**Figure 6:**
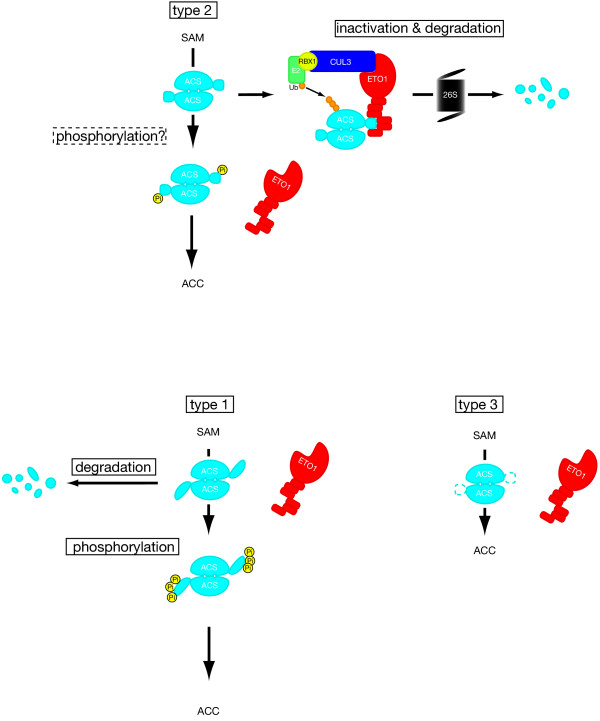
**Model for type-2-specific regulation of ACC synthases by ETO1**. ACC synthases and ETO1 proteins are shown in light blue and red, respectively. C-terminus of ACS is drawn as a small light blue circle or oval. Note that each pair of ACC synthases forms a dimer with shared active site (two pockets per dimer). Upper panel:, type 2 ACS dimer interacts with ETO1. As a result, the ACS is inhibited for enzymatic activity and targeted for proteasome-dependent degradation. Yet unknown modification (possibly phosphorylation (yellow circles)) of the C-termini of type 2 ACS may inhibit interaction between ACS and ETO1, resulting in ACC production. Lower panel (left) type 1: Although ETO1 does not interact with type 1 ACS dimer because type 1 ACS has a C-terminal tail (blue oval) not suitable for the interaction with ETO1, it is also degraded with yet unidentified mechanism. Triple phosphorylation of the C-termini by MAPK stabilizes type 1 ACS dimer and the dimer produces ACC from SAM. Lower panel (right), type 3: ETO1 does not interact with type 3 ACS dimer because it lacks the specific C-terminal tail required for the interaction with ETO1.

Little is known about the endogenous regulators of ethylene biosynthesis in the control of plant development, response to stress or fruit ripening. Because tomato has at least one ETO1 ortholog [LeEOL1; HY and JRE, unpublished], a family of ACS isozymes and many ethylene-related phenotypes, we tested whether ETO1 could interact with and inhibit members of the ACC synthase family from tomato. We showed that ETO1 specifically interacts with two type 2 ACC synthases, AtACS5 and LE-ACS3, but not with LE-ACS2 (type 1) or LE-ACS4 (type 3) in the yeast two-hybrid system. Furthermore, we demonstrated that overexpression of the *ETO1 *transgene posttranscriptionally suppresses the activity of LE-ACS3 in a heterologous plant. These results suggest that type 2 ACC synthases represent a group that specifically interact with and are inhibited by ETO1 family proteins (Fig. 6). Whether the inhibitory effect of the ETO1 family *in planta *is limited to type 2 is still unknown; we have not yet checked the interaction of ETO1 with LE-ACS2 and LE-ACS4 *in planta*. In the yeast two-hybrid system, the interaction of ETO1 was obviously restricted to type 2 isozymes. However, *in planta*, AtMPK6-dependent phosphorylation of AtACS6 (a type 1 ACS) can stabilize this isozyme and increase ethylene biosynthesis [17]. Phosphorylation of such kind of modification of type 1 ACC synthases may confer an ability to interact with ETO1 [52]. Also, a calcium-dependent phosphorylation of the serine residue within the RLSF motif of LE-ACS2 [40], or any proteolytic cleavage [27,40] may change the C-terminal conformation and enable interaction with the ETO1 family. These possibilities may be elucidated by coimmunoprecipitation experiments using antibodies that can distinguish phosphorylated from nonphosphorylated forms of type 1 ACS, or pull-down assay using type-1-specific C-terminal peptide. In contrast, type 3 ACC synthases does not contain this potential phosphorylation site, suggesting that they may not be similarly regulated. The Ctr^- ^phenotype of plants constitutively expressing the C-terminally truncated version of AtACS5 [41] supports this hypothesis.

Another question is whether any other members of the ETO1 family interact with other types of ACC synthases. In the yeast two-hybrid system, both of the two paralogs of ETO1, EOL1 and EOL2, showed an interaction with ACC synthases from tomato similar to that of ETO1 (i.e., they interacted only with type 2 ACS but not with type 1 or type 3; data not shown). This suggests that type-2-specific regulation may be common to other members of the ETO1 family at least in yeast cells. However, a possibility that any EOL proteins may interact with and inhibit ACC synthases other than type 2 isozymes modified *in planta *is still not excluded.

### C-Terminal sequence specific to type 2 ACS is a target of ETO1

The C-terminal amino acid sequence specific to type 2 ACS (i.e., the WVFRLSF followed by the R/D/E-rich region), is a target of ETO1. In addition, mutations found within the RLSF to the R/D/E-rich region of AtACS5 of *eto2-1 *and the WVF motif of AtACS9 of the *eto3 *mutant [36,38] strongly implicate the importance of this amino acid sequence. Among molecular lesions of *Arabidopsis eto1 *mutations identified so far, *eto1-1*, which lacks only the last TPR motif, has a phenotype similar to the other alleles, and a C-terminal deletion mutant of ETO1 (tETO1) impaired in its last TPR motif failed to interact with AtACS5 in the yeast two-hybrid assay [41]. These results indicate that TPR motifs of ETO1 are essential for interaction with AtACS5.

TPR motifs are involved in various protein-protein interactions. Generally, the interaction between TPR domains and their target peptide are strictly limited by both electrostatic and hydrophobic interactions [53]. In this regard, type-2-specific C-termini of ACC synthases have some features required for the strict interaction with the TPR of ETO1. The crystal structure of LE-ACS2 has been determined, and the C-terminal tail of LE-ACS2 seems to protrude from the surface of the ACS monomer and dimer [54]. In the dimeric form (head-to-tail orientation) of LE-ACS2, N-terminal residues 11–19 make contact with the C-terminal helix H14 just before the C-terminal tail. The significance of the interaction between the N- and the C-termini is not clear but may be important for conformation stabilization and catalysis, as suggested by biochemical studies [50]. The WVF motif is likely located outside this N- and C-terminal interaction region and may be important for formation of the interacting surface with ETO1. Crystal structure analysis of the ETO1-ACS complex would reveal the significance of the WVF motif. Also, an isozyme of type 2 ACS, LE-ACS8, does not possess the precise WVF sequence. Instead, it contains WGF, which is very closely related to WVF (Fig. 3). In contrast to the *eto3 *version of AtACS9, in which the valine residue of the WVF motif was altered to the charged aspartic acid residue, the glycine residue in the C-terminus of LE-ACS8 is a neutral amino acid. Therefore, it is likely that LE-ACS8 may also interact with ETO1 as do other type 2 isozymes.

### Posttranslational regulation of ethylene biosynthesis

We have identified many orthologous sequences of *ETO1 *from other plant species including tomato, suggesting that the ETO1-based regulatory system is common among the plant kingdom. In the present study, we showed that *Arabidopsis *ETO1 interacts with and posttranscriptionally regulates LE-ACS3, a type 2 ACS, in a heterologous plant, tomato. This indicates that the ETO1 protein family is a common component in the negative regulation of ethylene biosynthesis in plants. Given that ETO1-homologous sequences are found in all plants but not in animals or prokaryotes, this system of regulation of ethylene may have developed early in plant evolution. ACC synthases are similar to the subgroup I family of pyridoxal 5-phosphate (PLP)-dependent aminotransferases [55]. Most of the similarity between these two families is found around the active sites, while the target of ETO1 lies in the C-terminus of ACS, which is outside the conserved region. It is an intriguing question as to how early plants acquired this unique mechanism to regulate ethylene biosynthesis.

Finally, one may ask why only type 2 ACC synthases are regulated by ETO1. It is imperative for plants to have ethylene synthesized in a timely manner. For instance, a remarkable phenotype of all *eto *mutants was observed in the etiolated seedling stage, suggesting that production of ethylene is regulated by ETO1 during germination. Although ethylene evolution is important for germination, seedlings with excessive level of ethylene for an extended time would continue to exhibit phenotype similar to that of the triple response, which is normally disappeared after germination. Also ethylene is important to respond to various stresses. To avoid unnecessary overproduction of ethylene in physiological and developmental processes, a tightly regulated system consisted of the type 2 ACC synthases and ETO1 protein family together with yet unknown system (for instance, AtMPK6-AtACS6 system) must have been evolved in plants to timely control ethylene biosynthesis until this important growth regulator and stress phytohormone is needed.

## Conclusion

In this study, we elucidated the substrate specificity of ETO1 protein. We showed the interaction between ETO1 and ACS protein family is restricted to type 2 ACS isozymes which possess specific C-terminal amino acid sequences. Our data that ETO1 suppress auxin-induced ethylene evolution through induction of *LE-ACS3 *also show that *Arabidopsis *ETO1 can regulate type 2 ACS in a heterologous plant.

## Methods

### Plant material

Plants of wild type and transformed tomato (*L. esculentum *cv. Shu-gyoku) were grown in greenhouses under standard conditions. For transformation, seedlings were grown on medium supplemented with 1/2 × Murashige and Skoog (MS) minimal salts.

### Transformation of tomato

Tomato hypocotyls or cotyledons precultured on MS medium containing 1 mg/l NAA, 0.5 mg/l BA, and 3% sucrose were transformed by an *Agrobacterium*-mediated procedure [56]. Full-length *ETO1 *cDNA was introduced in the sense orientation between the CaMV 35S promoter and Nos terminator of the pROK2 vector harbouring *NPT2 *gene as a selectable marker [41]. Transformed plants were confirmed by PCR amplification using specific primers for the 35S promoter and the *ETO1 *cDNA.

### Nucleic acid analysis

Genomic DNA was isolated using Isoplant II (Nippon Gene). Total RNA was extracted using RNeasy Plant Mini Kit (Qiagen). Transcription levels of *ETO1 *were analyzed by reverse RT-PCR analysis of total RNA. RNA (1 μg) was used in each RT-PCR following the manufacturer's protocol (Titan One Tube RT-PCR System, Roche Diagnostics). Gene-specific primers for *ETO1 *were used, and *GAPDH *was used as an internal control. Both of the primer sets were added to the same tube. PCR amplification was performed for a first round of 10 cycles as follows: denaturation at 94°C for 30 sec, annealing at 55°C for 30 sec, and extension at 68°C for 45 sec. Then the second round of cycles was performed as follows: denaturation at 94°C for 30 sec, annealing at 55°C for 30 sec, and extension at 68°C for 45 sec + 5/cycle sec. The final extension was carried out at 68°C for 7 min, and the reaction was then stopped at 4°C. For Northern blot analysis, total RNA (10 μg) was used for each lanes and blotted onto positively charged nylon membranes (Roche Diagnostics). DIG-labeled RNA probe for *LE-ACS3 *was synthesized following the manufacturer's protocol (Roche Diagnostics) and detected by CDP-Star (Roche Diagnostics).

### Yeast two-hybrid assay

Yeast two-hybrid assays were performed as described previously [41]. *ETO1 *cDNA was cloned into the pAS2 vector. cDNAs for *LE-ACS2*, *LE-ACS3*, and *LE-ACS4 *were synthesized by RT-PCR using SuperScriptII (Invitrogen) reverse transcriptase and *Pfu*Turbo DNA polymerase (Stratagene) and cloned into pACT2. Deletion or chimeric mutants of *LE-ACS2 *and *LE-ACS3 *were constructed by PCR using *Pfu*Turbo DNA polymerase. A quantitative liquid assay for β-galactosidase activity was performed according to the manufacturer's instructions (BD Biosciences). Each experiment was repeated at least three times using independent clones.

### Measurement of ethylene biosynthesis

Etiolated seedlings (1 per vial) of T_2 _homozygous lines 14-1-H2 (*ETO1*/*ETO1*) and 14-1-H4 (*wt*/*wt*) were grown in vials (10-mm diameter, 75-mm length with a rubber septum) containing 1 g of sea sand (20–35 mesh, Wako Chemicals) and 0.5 ml of water at 28°C in the dark. After 6 d, 2,4-D (in 1% ethanol) was added to the final concentration of 100 μM and seedlings were incubated under the same conditions. On the next day, the accumulated ethylene was measured using a gas chromatograph (Model GC-7A, Shimadzu) equipped with an active alumina column (60/80 mesh, 3 mm × 1.5 m) and FID. A 0.5-ml volume of each sample from the headspace was injected onto the column. Eight individuals were used for each treatment. Ethylene production was calculated as pL· seedling^-1^· hr^-1^.

## Authors' contributions

HY conceived of the study, arranged the funding for the project, made transgenic tomato, analyze their phenotypes and expression of the *ETO1 *transgene, made the two-hybrid vectors with mutant versions of ACS, and carried out all the yeast two-hybrid assays. MN measured ethylene evolution of tomatoes and carried out the Northern hybridization of *LE*-*ACS3*. KS participated in genotyping and maintenance of the transgenic plants. KLCW made the two-hybrid vectors with *ETO1*, participated in discussion and helped to draft the manuscript. JRE participate in discussion, and helped to draft the manuscript. All the authors read and approved the final manuscript.

## References

[B1] Johnson PR, Ecker JR (1998). The ethylene gas signal transduction pathway: a molecular perspective. Annu Rev Genet.

[B2] Wang KLC, Li H, Ecker JR (2002). Ethylene biosynthesis and signaling networks. Plant Cell.

[B3] Chang C, Kwok SF, Bleecker AB, Meyerowitz EM (1993). *Arabidopsis *ethylene-response gene *ETR1*: similarity of product to two-component regulators. Science.

[B4] Hua J, Chang C, Sun Q, Meyerowitz EM (1995). Ethylene insensitivity conferred by *Arabidopsis ERS *gene. Science.

[B5] Hua J, Sakai H, Nourizadeh S, Chen QG, Bleecker AB, Ecker JR, Meyerowitz EM (1998). *EIN4 *and *ERS2 *are members of the putative ethylene receptor gene family in *Arabidopsis*. Plant Cell.

[B6] Hua J, Meyerowitz EM (1998). Ethylene responses are negatively regulated by a receptor gene family in *Arabidopsis thaliana*. Cell.

[B7] Hirayama T, Kieber JJ, Hirayama N, Kogan M, Guzman P, Nourizadeh S, Alonso JM, Dailey WP, Dancis A, Ecker JR (1999). RESPONSIVE-TO-ANTAGONIST1, a Menkes/Wilson disease-related copper transporter, is required for ethylene signaling in *Arabidopsis*. Cell.

[B8] Kieber JJ, Rothenberg M, Roman G, Feldmann KA, Ecker JR (1993). *CTR1*, a negative regulator of the ethylene response pathway in *Arabidopsis*, encodes a member of the Raf family of protein kinases. Cell.

[B9] Clark KL, Larsen PB, Wang X, Chang C (1998). Association of the *Arabidopsis *CTR1 Raf-like kinase with the ETR1 and ERS ethylene receptors. Proc Natl Acad Sci USA.

[B10] Alonso JM, Hirayama T, Roman G, Nourizadeh S, Ecker JR (1999). EIN2, a bifunctional transducer of ethylene and stress responses in *Arabidopsis*. Science.

[B11] Chao Q, Rothenberg M, Solano R, Roman G, Terzaghi W, Ecker JR (1997). Activation of the ethylene gas response pathway in *Arabidopsis *by the nuclear protein ETHYLENE-INSENSITIVE3 and related proteins. Cell.

[B12] Solano R, Stepanova A, Chao Q, Ecker JR (1998). Nuclear events in ethylene signaling: a transcriptional cascade mediated by ETHYLENE-INSENSITIVE3 and ETHYLENE-RESPONSE-FACTOR1. Genes Dev.

[B13] Alonso JM, Stepanova AN, Leisse TJ, Kim CJ, Chen H, Shinn P, Stevenson DK, Zimmerman J, Barajas P, Cheuk R, Gadrinab C, Heller C, Jeske A, Koesema E, Meyers CC, Parker H, Prednis L, Ansari Y, Choy N, Deen H, Geralt M, Hazari N, Hom E, Karnes M, Mulholland C, Ndubaku R, Schmidt I, Guzman P, Aguilar-Henonin L, Schmid M, Weigel D, Carter DE, Marchand T, Risseeuw E, Brogden D, Zeko A, Crosby WL, Berry CC, Ecker JR (2003). Genome-wide insertional mutagenesis of *Arabidopsis thaliana*. Science.

[B14] Guo H, Ecker JR (2003). Plant responses to ethylene gas are mediated by SCF^EBF1/EBF2^-dependent proteolysis of EIN3 transcription factor. Cell.

[B15] Potuschak T, Lechner E, Parmentier Y, Yanagisawa S, Grava S, Koncz C, Genschik P (2003). EIN3-dependent regulation of plant ethylene hormone signaling by two *Arabidopsis *F box proteins: EBF1 and EBF2. Cell.

[B16] Ouaked F, Rozhon W, Lecourieux D, Hirt H (2003). A MAPK pathway mediates ethylene signaling in plants. EMBO J.

[B17] Liu Y, Zhang S (2004). Phosphorylation of 1-aminocyclopropane-1-carboxylic acid synthase by MPK6, a stress-responsive mitogen-activated protein kinase, induces ethylene biosynthesis in *Arabidopsis*. Plant Cell.

[B18] Ecker JR (2004). Reentry of the Ethylene MPK6 Module. Plant Cell.

[B19] Roman G, Lubarsky B, Kieber JJ, Rothenberg M, Ecker JR (1995). Genetic analysis of ethylene signal transduction in *Arabidopsis thaliana *: five novel mutant loci integrated into a stress response pathway. Genetics.

[B20] Larsen PB, Chang C (2001). The *Arabidopsis eer1 *mutant has enhanced ethylene responses in the hypocotyl and stem. Plant Physiol.

[B21] Sato T, Theologis A (1989). Cloning the mRNA encoding 1-aminocyclopropane-1-carboxylate synthase, the key enzyme for ethylene biosynthesis in plants. Proc Natl Acad Sci USA.

[B22] Nakajima N, Nakagawa N, Imaseki H (1990). Molecular size of wound-induced 1-aminocyclopropane-1-carboxylate synthase from *Cucurbita maxima *Dutch. and change of translatable mRNA of the enzyme after wounding. Plant Cell Physiol.

[B23] Hamilton AJ, Bouzayen M, Grierson D (1991). Identification of a tomato gene for the ethylene-forming enzyme by expression in yeast. Proc Natl Acad Sci USA.

[B24] Spanu P, Reinhardt D, Boller T (1991). Analysis and cloning of the ethylene-forming enzyme from tomato by functional expression of its mRNA in *Xenopus laevis *oocytes. EMBO J.

[B25] Van der Straeten D, Rodrigues-Pousada RA, Villarroel R, Hanley S, Goodman HM, Van Montagu M (1992). Cloning, genetic mapping, and expression analysis of an *Arabidopsis thaliana *gene that encodes 1-aminocyclopropane-1-carboxylate synthase. Proc Natl Acad Sci USA.

[B26] Kende H (1993). Ethylene biosynthesis. Annu Rev Plant Physiol Plant Mol Biol.

[B27] Yamagami T, Tsuchisaka A, Yamada K, Haddon WF, Harden LA, Theologis A (2003). Biochemical diversity among the 1-amino-cyclopropane-1-carboxylate synthase isozymes encoded by the *Arabidopsis *gene family. J Biol Chem.

[B28] Moeder W, Barry CS, Tauriainen AA, Betz C, Tuomainen J, Utriainen M, Grierson D, Sandermann H, Langebartels C, Kangasjarvi J (2002). Ethylene synthesis regulated by biphasic induction of 1-aminocyclopropane-1-carboxylic acid synthase and 1-aminocyclopropane-1-carboxylic acid oxidase genes is required for hydrogen peroxide accumulation and cell death in ozone-exposed tomato. Plant Physiol.

[B29] Yang SF, Hoffman NE (1984). Ethylene biosynthesis and its regulation in higher plants. Annu Rev Plant Physiol.

[B30] Mattoo AK, Suttle JC (1991). The Plant Hormone Ethylene.

[B31] Abeles FB, Morgan PW, Salveit ME (1992). Ethylene in Plant Biology.

[B32] Nakatsuka A, Murachi S, Okunishi H, Shiomi S, Nakano R, Kubo Y, Inaba A (1998). Differential expression and internal feedback regulation of 1-aminocyclopropane-1-carboxylate synthase, 1-aminocyclopropane-1-carboxylate oxidase, and ethylene receptor genes in tomato fruit during development and ripening. Plant Physiol.

[B33] Peck SC, Kende H (1998). Differential regulation of genes encoding 1-aminocyclopropane-1-carboxylate (ACC) synthase in etiolated pea seedlings: effects of indole-3-acetic acid, wounding, and ethylene. Plant Mol Biol.

[B34] Spanu P, Grosskopf DG, Felix G, Boller T (1994). The apparent turnover of 1-aminocyclopropane-1-carboxylate synthase in tomato cells regulated by protein phosphorylation and dephosphorylation. Plant Physiol.

[B35] Oetiker JH, Olson DC, Shiu OY, Yang SF (1997). Differential induction of seven 1-aminocyclopropane-1-carboxylate synthase genes by elicitor in suspension cultures of tomato (*Lycopersicon esculentum*). Plant Mol Biol.

[B36] Vogel JP, Woeste KE, Theologis A, Kieber JJ (1998). Recessive and dominant mutations in the ethylene biosynthetic gene *ACS5 *of *Arabidopsis*confer cytokinin insensitivity and ethylene overproduction, respectively. Proc Natl Acad Sci USA.

[B37] Woeste KE, Ye C, Kieber JJ (1999). Two Arabidopsis mutants that overproduce ethylene are affected in the posttranscriptional regulation of 1-aminocyclopropane-1-carboxylic acid synthase. Plant Physiol.

[B38] Chae HS, Faure F, Kieber JJ (2003). The *eto1*, *eto2*, and *eto3 *mutations and cytokinin treatment increase ethylene biosynthesis in *Arabidopsis *by increasing the stability of ACS protein. Plant Cell.

[B39] Liang X, Shen NF, Theologis A (1996). Li^+^-regulated 1-aminocyclopropane-1-carboxylate synthase gene expression in *Arabidopsis thaliana*. Plant J.

[B40] Tatsuki M, Mori H (2001). Phosphorylation of tomato 1-aminocyclopropane-1-carboxylic acid synthase, LE-ACS2, at the C-terminal region. J Biol Chem.

[B41] Wang KLC, Yoshida H, Lurin C, Ecker JR (2004). Regulation of ethylene gas biosynthesis by the *Arabidopsis *ETO1 protein. Nature.

[B42] Guzman P, Ecker JR (1990). Exploiting the triple response of *Arabidopsis *to identify ethylene-related mutants. Plant Cell.

[B43] Larsen PB, Cancel JD (2004). A recessive mutation in the RUB1-conjugating enzyme, *RCE1*, reveals a requirement for RUB modification for control of ethylene biosynthesis and proper induction of *basic chitinase *and *PDF1.2 *in *Arabidopsis*. Plant J.

[B44] Bostick M, Lochhead SR, Honda A, Palmer S, Callis J (2004). Related to ubiquitin 1 and 2 are redundant and essential and regulate vegetative growth, auxin signaling, and ethylene production in *Arabidopsis*. Plant Cell.

[B45] Oeller PW, Lu MW, Taylor LP, Pike DA, Theologis A (1991). Reversible inhibition of tomato fruit senescence by antisense RNA. Science.

[B46] Lincoln JE, Campbell AD, Oetiker J, Rottmann WH, Oeller PW, Shen NF, Theologis A (1993). *LE-ACS4*, a fruit ripening and wound-induced 1-aminocyclopropane-1- carboxylate synthase gene of tomato (*Lycopersicon esculentum*). Expression in *Escherichia coli*, structural characterization, expression characteristics, and phylogenetic analysis. J Biol Chem.

[B47] Yip WK, Moore T, Yang SF (1992). Differential accumulation of transcripts for four tomato 1-aminocyclopropane-1-carboxylate synthase homologs under various conditions. Proc Natl Acad Sci USA.

[B48] Zarembinski TI, Theologis A (1994). Ethylene biosynthesis and action: a case of conservation. Plant Mol Biol.

[B49] Tsuchisaka A, Theologis A (2004). Heterodimeric interactions among the 1-amino-cyclopropane-1-carboxylate synthase polypeptides encoded by the *Arabidopsis *gene family. Proc Natl Acad Sci USA.

[B50] Li N, Mattoo AK (1994). Deletion of the carboxyl-terminal region of 1-aminocyclopropane-1-carboxylic acid synthase, a key protein in the biosynthesis of ethylene, results in catalytically hyperactive, monomeric enzyme. J Biol Chem.

[B51] Olson DC, Oetiker JH, Yang SF (1995). Analysis of *LE-ACS3*, a 1-aminocyclopropane-1-carboxylic acid synthase gene expressed during flooding in the roots of tomato plants. J Biol Chem.

[B52] Kim CY, Liu Y, Thorne ET, Yang H, Fukushige H, Gassmann W, Hildebrand D, Sharp RE, Zhang S (2003). Activation of a stress-responsive mitogen-activated protein kinase cascade induces the biosynthesis of ethylene in plants. Plant Cell.

[B53] Scheufler C, Brinker A, Bourenkov G, Pegoraro S, Moroder L, Bartunik H, Hartl FU, Moarefi I (2000). Structure of TPR domain-peptide complexes: critical elements in the assembly of the Hsp70-Hsp90 multichaperone machine. Cell.

[B54] Huai Q, Xia Y, Chen Y, Callahan B, Li N, Ke H (2001). Crystal structures of 1-aminocyclopropane-1-carboxylate (ACC) synthase in complex with aminoethoxyvinylglycine and pyridoxal-5'-phosphate provide new insight into catalytic mechanisms. J Biol Chem.

[B55] Mehta PK, Hale TI, Christen P (1993). Aminotransferases: demonstration of homology and division into evolutionary subgroups. Eur J Biochem.

[B56] Nagata M, Mori H, Tabei Y, Sato T, Hirai M, Imaseki H (1995). Modification of tomato fruit ripening by transformation with sense or antisense chimeric 1-aminocyclopropane-1-carboxylate synthase genes. Acta Horticulture.

